# Serum vitamin D levels and acute kidney injury: a systemic review and meta-analysis

**DOI:** 10.1038/s41598-022-24560-4

**Published:** 2022-11-27

**Authors:** Huanran Zhang, Yan Jiang, Nan Shi, Yuan-Qiang Lu

**Affiliations:** 1 Department of Emergency Medicine, School of Medicine, The First Affiliated Hospital, Zhejiang University, Hangzhou, Zhejiang 310003 People’s Republic of China; 2 Key Laboratory of Diagnosis and Treatment of Aging and Physic-Chemical Injury Diseases of Zhejiang Province, School of Medicine, The First Affiliated Hospital, Zhejiang University, Hangzhou, Zhejiang People’s Republic of China; 3 Kidney Disease Center, School of Medicine, The First Affiliated Hospital, Zhejiang University, Hangzhou, People’s Republic of China

**Keywords:** Acute kidney injury, Prognostic markers, Nutrition

## Abstract

Numerous researches have evaluated the prevalence and clinical outcome of vitamin D deficiency in patients with chronic kidney disease (CKD) and end-stage renal disease (ESRD). But the quantitative vitamin D status in acute kidney injury (AKI) patients and its relationship with prognosis remains controversial. We conducted this systemic review and meta-analysis to assess the quantitative difference of vitamin D status, including serum 25(OH) D and 1,25(OH)2D levels, between AKI patients and non-AKI controls, and further explore whether vitamin D status can be clearly correlated with the mortality of AKI. Major databases, including PubMed, Web of Science and EBSCO, were searched until 1st September 2021. All published observational studies related to vitamin D and AKI According to predefined inclusion and exclusion criteria were extracted. Meta-analyses were performed using Review Manager 5.3.5. Four studies including five cohorts were included with a total of 413 patients. The serum 25(OH)D levels showed no statistically significant difference in AKI patients and non-AKI controls. On the other hand, the serum 1,25(OH)2D levels were significant lower in AKI patients than in non-AKI controls (MD =  − 17.79, 95% CI =  − 32.73 to − 2.85, *P* = 0.02). As for the relationship between serum vitamin D status and AKI patients’ mortality, we were unable to give a consistent conclusion based on current limited and conflict study results. Our meta-analysis suggested that serum 1,25(OH)2D levels, rather than 25(OH)D, is significantly lower in AKI patients. The relationship between vitamin D status and clinical outcome of AKI remains controversial based on current evidence. Future comprehensive studies are required to confirm these relations and to elucidate potential mechanisms.

## Introduction

Acute kidney injury (AKI) is a heterogeneous group defined as a sudden decrease in glomerular filtration rate (GFR), an increase in serum creatinine concentration (SCC) or oliguria. 10% AKI patients require kidney replacement therapy (KRT). Although KRT has been widely used, the mortality of critically ill patients with AKI is still relatively high. the mortality in whom remains approximately 50%^1^ In high-income countries, half AKI is hospital-acquired, sepsis, drugs or invasive procedures maybe the main causes; Well in low- to middle-income countries around 77% of AKI is community-acquired, the predominant causes of AKI are shown as infections and hypovolaemic shock. Globally, 60% of patients with AKI are male^2^. Observational studies suggest that the elevated mortality in AKI patients cannot be explained solely by the increased complications. Kidney injury itself is an independent risk factor of adverse events^3,4^. AKI occurs means the main function of the kidneys(maintaining homeostasis, fluid homeostasis, acid–base homeostasis et al.) loss, further complications such as acidosis, uraemic toxins, volume overload^1^, electrolyte disorders and systemic inflammation, affects most organ systems of the body. The first step in managing AKI is to determine and treat the cause because AKI is not a disease but rather a loose collection of syndromes. Other methods include attention to fluid status, haemodynamic management, stop all potentially nephrotoxic agents, KRT and so on^2^ More then that kidney is also an important endocrine organ, kidney injury is often accompanied by disorders of endocrine and mineral metabolism, including vitamin D system dysfunction^5^.

In humans, > 95% of systemic vitamin D3 (D3) is synthesized in the skin under UVB irradiation^6^, after absorption of UVB energy by the B ring of 7DHC leading to its opening to produce the pre-vitamin D3, then undergoes a temperature-dependent isomerization to D3^7,8^. In the classical pathway, D3 is first converted into 25(OH)D in the liver at C25 by 25-hydroxylase(CYP2R1 or CYP27A1)and hydroxylated at C1αin the kidney or peripheral tissues expressing 1-αhydroxylase(CYP27B1)^6^ as well as the steroidogenic enzyme cytochrome P450scc (CYP11A1) to 1,25(OH)_2_D^8–11^ 1,25(OH)_2_D is the most extensively characterized active naturally occurring D3 metabolite. In addition to regulating calcium homeostasis, has pleiotropic activities such as immunomodulatory properties, inflammatory processes, regulation of the global metabolic and endocrine homeostasis and functions of the cardiovascular system^12,13^. The half-life of D3, 25(OH)D, 1,25(OH)2D is 24 h, 3 weeks and 4 h, respectively^14^. Thus, the nutritional status of vitamin D in the published literatures and clinical practice usually refers to serum 25 (OH) D levels. 1,25(OH)2D is the main bioactive metabolite of vitamin D and can exert the greatest physiological effect^13^. However, it is less often measured in the clinical practice due to its short half-life. In addition to regulating calcium and phosphorus metabolism and maintaining bone and mineral homeostasis, vitamin D also plays important role in tumors, cardiovascular system, autoimmune system and endocrine system^15–17^.

Hydroxylation of 25(OH)D into 1,25(OH)2D takes place mainly in the inner mitochondrial membrane of the renal proximal tubule epithelium, which is quite vulnerable during AKI. This leads to our reasonable assumptions that vitamin D status, especially 1,25(OH)2D levels, may be decreased in AKI patients. Numerous researches have evaluated the prevalence of vitamin D deficiency, typically reported as 25(OH)D, in patients with chronic kidney disease (CKD) and end-stage renal disease (ESRD), and it is associated with mortality^3,4,18,19^. But relative fewer studies focused on the correlation between AKI and vitamin D status, including the 25(OH) D and 1,25(OH)2D levels, and its relationship with prognosis remains controversial. Therefore, we conducted this systemic review and meta-analysis to assess the serum 25(OH) D and 1,25(OH)2D levels in AKI patients, and further explore whether vitamin D status Records Identified can be clearly correlated with the mortality of AKI.

## Methods

### Study selection

We searched the PubMed, Web of Science and EBSCO for studies published up to 1st September 2021. Following search terms: vitamin D, 25(OH) vitamin D, 25(OH) D, 1,25(OH)2 vitamin D, 1,25(OH)2D, AKI, acute kidney injury, acute renal injury were used. ‘OR’ was used as the set operator to combine different sets of results. Furthermore, references of relevant reviews and included articles were searched manually to identify additional studies.

Studies were included if they met the following criteria:1)Predefined AKI; 2) Adult patients with AKI (aged ≥ 18 years old, human beings); 3) The study should include quantitative data of serum 25(OH)D and /or 1,25(OH)_2_D levels; 4) For duplicate or overlapping data, including only the most recent or comprehensive data; 5) language was restricted to English only.

Exclusive criteria were as follows:1) studies that conducted in animals or children;2) a document in which the required data cannot be extracted due to unclear or erroneous data;3)review articles or case reports; 4) use of medications containing vitamin D or calcium.

**Figure 1:**
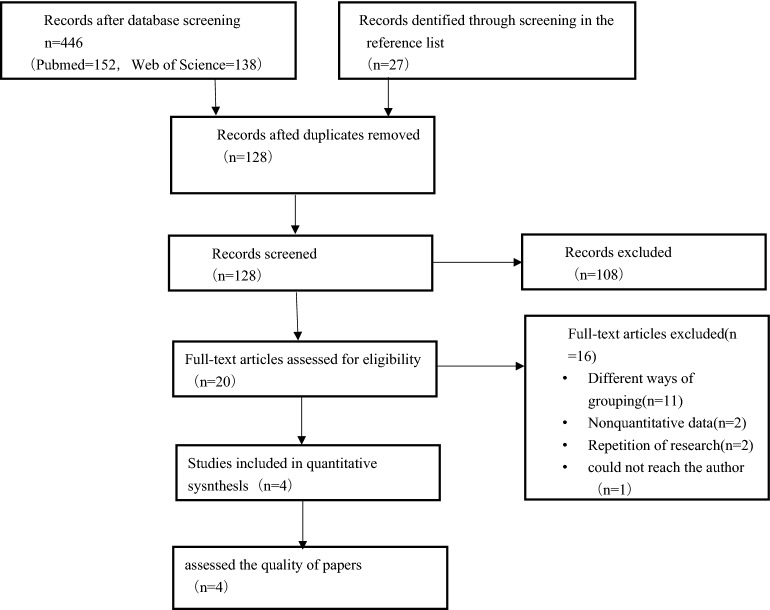
Flow diagram of study selection process.

Two investigators (HRZ and YJ) independently reviewed all identified articles for eligibility using the above criteria. The titles and abstracts of the identified articles were reviewed, and those deemed ineligible were excluded. The full text of the remainder of the articles was retrieved and reviewed. Discrepancies on whether to include a study were resolved by discussion. See Fig. 1.

### Data extraction

The data extracted from each included study by two separate reviewers (HRZ and YJ) included the authors, year of publication, design of the trial, sex, sample size, follow-up, t1. Discrepancies in data abstraction were resolved by discussion. The data were reviewed to identify duplicate studies and duplicate reporting of populations. Only the most comprehensive studies were retained.

### Quality assessment

Two investigators (HRZ and YJ) independently evaluated the methodological quality of the included studies according to the Newcastle–Ottawa scale (NOS), which including selection (0–4 points), comparability (0–2 points) and outcome (0–3 points). Discrepancies on quality assessment were resolved by discussion. See Table 1.

**Table 1:** Quality assessment of included studies (Newcastle–Ottawa Scale).

Author	Representativeness of the exposed cohort	Selection of the non exposed cohort	Ascertainment	Outcome was not present at start of study	Select the most important factor	Indicate specific control for a second important factor	Assessment of outcome	Follow-up time	Adequacy of follow-up	Quality
Anitha Vijayan, 2015	*		*	*	*	*	*	*	*	High
Lai, 2013	*	*	*	*	*	*	*	*		High
David E. Leaf, 2013	*	*	*	*	*	*	*	*	*	High
Murat Gunay, 2019	*		*	*	*	*	*	*	*	High

*mean the article meets the entry of Newcastle-Ottawa Scale and gets 1 score.

### Statistical analysis

All statistical analyses were performed using the REVIEW MANAGER 5.3 statistical software (Cochrane Collaboration, Oxford, UK). Quantitative vitamin D values in 3 of the 4 included articles were recorded in the form of mean ± standard deviation (SD), while the remaining one recorded in the form of medium (25–75% IQR). To unify data, we transformed medium (25–75% IQR) to mean ± SD. Lai's study included two control groups, age- and gender-matched healthy subjects(h) and critically ill patients without AKI(i). Therefore, we compared these two control groups with the experimental group (AKI) separately as two cohorts, while AKI group’s sample size was divided half in each cohort. For continuous variables, we calculated the pooled Mean Deference (MD) and 95% confidence intervals (CI) with the Inverse-Variance method using a random effects model. Heterogeneity was assessed by the I2 measure of inconsistency, statistically significant if I2 was > 50%. For all the outcomes a *P* < 0.05 was considered statistically significant. the final article included is showed in Table 2.

**Table 2:** Summary of characteristics of the included studies.

Reference	Country	Study design	Sample size	AKI group					Non-AKI group				
				Sample size	Age	Male(%)	25(OH) D(ng/ml)^a^	1,25(OH)_2_D(pg/ml)^b^	Sample size	Age	male sex%	25‐OH D(ng/ml)^a^	1,25(OH)_2_D(pg/ml)^b^
Anitha Vijayan, 2015	U.S.A	Prospective cohort study	46	34	51.9 ± 17.8	44.1	13.0 ± 6.3	42 ± 5.6	12	45.3 ± 16.9	41.7	29.2 ± 9.4	76.1 ± 5.3
Lai, 2013 h	China	Prospective cohort study	117	100	63.7 ± 18.8	73.5	13.9 ± 5.8	24.8 ± 22.0	17	56.1 ± 18.7	70.6	11.3 ± 3.6	35.9 ± 14.7
Lai, 2013 i	China	Prospective cohort study	113	100	63.7 ± 18.8	73.5	13.9 ± 5.8	24.8 ± 22.0	13	55.7 ± 16.3	69.2	12.9 ± 6.5	41.2 ± 16.5
David E. Leaf, 2013	U.S.A	Prospective cohort study	60	30	57 (50 − 64)	66.7	9.07 ± 8.56	16.29 ± 9.34	30	56 (45 − 61)	60.0	14.36 ± 10.12	25 ± 15.57
Murat Gunay, 2019	Turkey	Prospective cohort study	77	39	51.3	20	11.1 ± 9.3	–	38	38.5	52.6	11.3 ± 12.9	–

*a *p* = 0.11; *b *p* = 0.02.

## Results

### Study characteristics

The flow diagram for study selection is displayed in Fig. 1. A total of 446 records through searching the electronic database and 27 in the reference list articles identified. After removing duplicate publications, 128 studies were retrieved for title and abstract review. A total of 20 studies went further for full-text evaluation. 11 studies were excluded due to inconsistent study and grouping methods;2 were excluded because the report form cannot be used to calculate the results, 2 because repetition of research; 1 because we could not reach the author. We eliminated two overlapping data, including only the most recent or comprehensive data. Lai's study^20^ contains two control groups, healthy subjects(h) and critically ill patients without AKI(i), so it is divided into 2 cohorts(Halved the number of patients in the AKI group so that to comparison). Healthy subjects(h) subgroup inclusion criteria: age-and gender-matched healthy subjects, randomly obtained from among healthy patients of the health check-up center during the same period; Critically ill patients without AKI(i) subgroup: age, gender and Sequential Organ Failure Assessment (SOFA) score matched inpatient subjects during the same period. Finally, four studies with five cohorts fulfilled the inclusion criteria and were suitable for the analysis.

The characteristics of the included studies are summarized in Table 1. Total sample size of four studies was 413. Methodological quality of the included studies using the NOS are presented in Table 2. All the enrolled articles showed moderate to high quality.

### Vitamin D values in AKI patients and non-AKI controls

**Figure 2:**
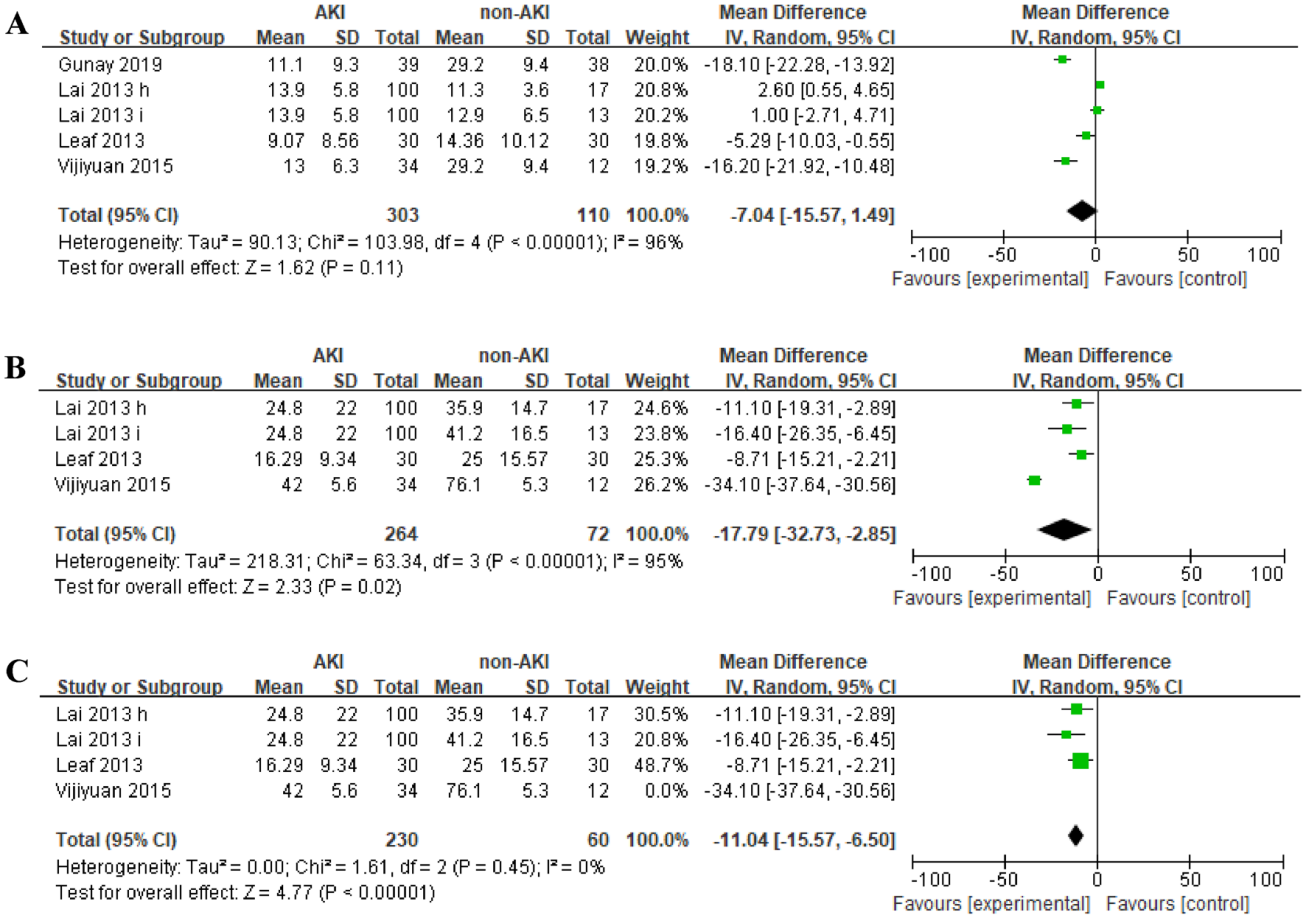
Forest plots of the effect size of the association between the incidence of AKI and serum (**A**) 25(OH)D levels (**B**) 1,25(OH)_2_D levels (**C**) 1,25(OH)_2_D levels removing Vijiyuan. et al.

To assess the quantitative difference of vitamin D status between AKI patients and non-AKI controls, five cohorts provided data on serum 25(OH)D level, and four on serum 1,25(OH)2D level. The average 25(OH)D levels in AKI patients was not significant different compared to non-AKI controls (MD =  − 7.04, 95% CI =  − 15.57 to 1.49, *P* = 0.11) (Fig. 2A), which showed significant heterogeneity (I^2^ = 96%, *p* < 0.0001). The average 1,25(OH)2D levels were significant lower in AKI patients than that in non-AKI controls (MD =  − 17.79, 95% CI =  − 32.73 to − 2.85, *P* = 0.02) (Fig. 2B), which also exhibited great heterogeneity (I^2^ = 95%, *p* < 0.0001). Since the result of the average 1,25(OH)2D levels in two groups was statistically significant, we further removed Vijiyuan’s study,significantly reduced this heterogeneity (I^2^ = 0, *p* = 0.45), without much impact on the outcome of 1,25(OH)2D levels (MD = − 11.04, 95% CI =  − 15.57 to  − 6.50, P < 0.01) (Fig. 2C).

We further performed a subgroup analysis based on two different control populations, which were divided into healthy subjects(h) subgroup and critically ill patients without AKI(i) subgroup, see Table 3.

**Table 3:** The number and means values of the participants in subgroups.

	Cohorts	25(OH)D (number/ means values)		1,25(OH)_2_D(number/ means values)	
		AKI	Non-AKI	AKI	Non-AKI
Health	Gunay, 2019	39/11.1	38/29.2	–	–
	Lai, 2013 h	100/13.9	17/11.3	100/24.8	17/35.9
	Vijiyuan, 2015	34/13.0	12/29.2	34/42.0	12/76.1
	Total	173/13.09	67/24.66	134/29.16	29/52.53
Ill	Lai, 2013 i	100/13.9	13/12.9	100/24.8	13/41.2
	Leaf, 2013	30/9.07	30/14.36	30/16.29	30/25.0
	Total	130/12.79	43/13.92	130/22.84	43/29.90
*P* value		0.11		0.02	

**Figure 3:**
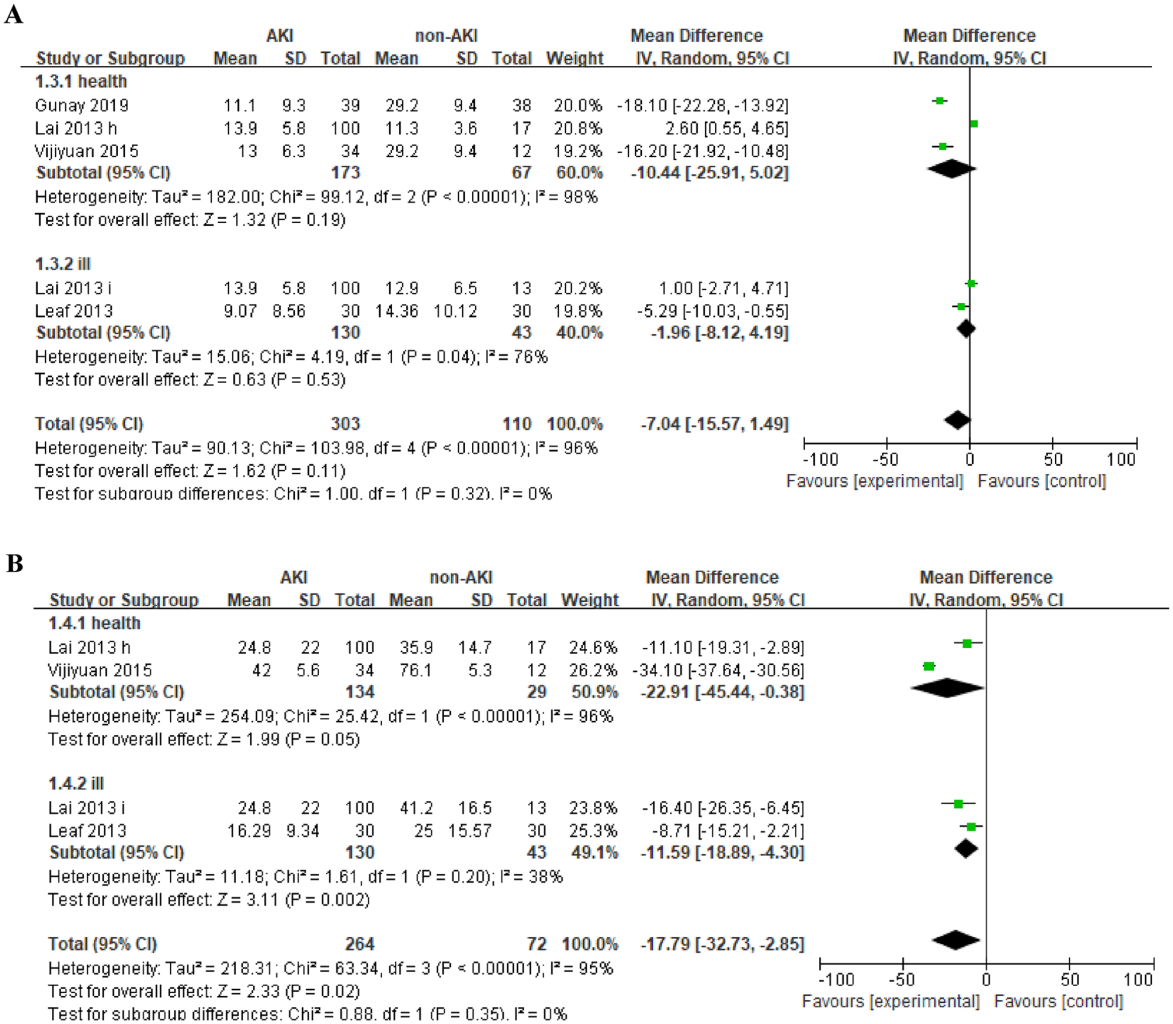
Forest plots of subgroup analysis of the relation between the incidence of AKI and serum Vitamin D in the different contrasts: (**A**) 25(OH)D levels (**B**). 1,25(OH)_2_D levels levels.

The serum 25(OH)D levels remained no significant difference in both subgroups. (Fig. 3A). The serum 1,25(OH)2D levels tend to be lower in AKI patients than in healthy subjects (MD =  − 22.91, 95% CI =  − 45.44 to − 0.38, *P* = 0.05) (Fig. 3B), but did not reach statistical difference. The serum 1,25 (OH)2 D levels were significant lower in AKI patients than critically ill patients without AKI (MD =  − 11.59, 95% CI =  − 18.89 to − 4.30, *P* = 0.02) (Fig. 3B).

### Relationship between vitamin D levels and prognosis of AKI

Of three included studies reporting AKI patients’ clinical outcome, only Vijayan^14^ provided quantitative data on vitamin D levels between the non-survivors and survivors among the AKI patients. Their study found that 1,25(OH)2D levels were significant higher in non-survivors than survivors (62 ± 41.4 pg/mL vs. 33.7 ± 24.2 pg/mL, *P* = 0.046), while 25(OH)D levels did not differ between two groups. However, higher levels of 1,25(OH)2D was not associated with mortality in AKI patients on multivariate regression analysis t after adjusting for age and APACHE II. Lai^20^ grouped the patients with AKI according to median serum vitamin D concentrations. They come to the conclusion there was no association between the serum vitamin D levels, both 25(OH)D and 1,25(OH)2D, and all-cause mortality in AKI patients. Similarly, Leaf reported that hospital mortality of AKI patients was not associated with 25(OH)D and 1,25(OH)2D after adjusting for age and serum creatinine.

Therefore, the relationship between vitamin D levels and mortality in AKI patients remains controversial. We are unable to give a consistent conclusion for this problem based on current limited and conflict study results. More comprehensive trials with sufficient power and longer follow-up duration are needed to draw a clear conclusion.

## Discussion

The kidney plays an important role in maintaining the stability of vitamin D system. Relationship between vitamin D status and CKD has been deeply researched^21^. Abundant evidence suggest that vitamin D supplementation can improve CKD patients’ prognosis^5^. However, less is known about the changes of vitamin D levels during AKI and its association with clinical outcome. In order to answer above problem, we conducted this systemic review and meta-analysis with great curiosity. Our study showed that AKI patients had a lower serum 1,25(OH)_2_D levels compared to non-AKI controls, while there was no difference in 25(OH) D levels between these two groups. Presently, we still cannot draw any definite conclusion for the association between vitamin D status and the mortality of AKI, due to limited published data. 1,25(OH)2D is formed mainly in the mitochondria of the proximal renal tubules by 1-α hydroxylase^22,23^. 1,25(OH)2D is the main bioactive metabolite of vitamin D and can exert the greatest physiological effect. But it is less detected in the clinical practice due to its short half-time. Instead,25 (OH) D is often used to reflect the vitamin status of the human body as it is convenient to detection and has a relative longer half-time. Renal tubules are vulnerable to injuries when AKI occurs, which may result in vitamin D system dysfunction. Therefore, we compared the serum levels both of 25 (OH) D and 1,25(OH)2D in patients with AKI to understand the true vitamin status in vivo.

The beneficial role of 25(OH)D and 1,25(OH)2D in CKD has been widely described in previous studies^21^. 1a-hydroxylase exists mainly in the renal proximal tubules. As the CKD progresses, the glomerulus gradually sclerosis, the tubule and interstitium gradually atrophy, and the amount of 1α hydroxylase decreases, resulting in 1,25(OH)2D deficiency. It is reported that vitamin D deficiency contributes to the burden of cardiovascular and total mortality in patients with CKD^24^. In a small study, Vitamin D supplementation reduced inflammatory cytokines such as IL-8, IL-6, and TNFα in chronic hemodialysis patients^25^. Vitamin D analogs have also been shown to have anti-inflammatory effects in patients with CKD^26^. The role of vitamin D in CKD has aroused our curiosity about its role in AKI.

Our study showed that AKI patients had a lower serum 1,25(OH)_2_ D levels compared to non-AKI controls. We speculate that when patients suffer from AKI, ischemia and necrosis of renal tubules, especially proximal tubules, may lead to sudden dysfunction or sudden decrease of 1a-hydroxylase, thus reducing the transformation to1,25(OH) _2_D^5^. Leaf studies^27^ found that fibroblast growth factor 23 (FGF-23) levels were significantly increased during AKI. FGF-23 could down-regulate the expression of renal 1α hydroxylase, thereby reducing the level of blood 1,25(OH) _2_D. Basic researches are needed to confirm these hypothesis.

Based on current evidence, our study cannot draw the clear conclusion on the relationship between vitamin D status and the mortality of AKI. Only Vijayan's study^14^ give the quantitative data on vitamin D levels between the non-survivors and survivors among the AKI patients. They found that the1,25(OH) _2_D levels were higher in non-survivors, but the 25(OH)D levels did not differ between groups. Their interpretation of this conclusion is that kidney is the main organ for active vitamin D production, but not the only organ. Macrophages in vivo can also stimulate the secretion of 1-α hydroxylase, leading to an increase in the level of 1,25(OH) 2D in the kidney^14^,Unlike Vijayan's study, Lai’s^20^ study and Leaf’s study^27^ did not give quantitative vitamin D data. They divided AKI patients into different groups according to medium vitamin D concentrations and concluded that no association exists between the serum vitamin D levels and all-cause mortality. Unfortunately, since the specific value of vitamin D levels between the death group and the survival group in their studies were not available, we could not conduct a meta-analysis for this question. Comprehensive studies are required to confirm these relations and to elucidate potential mechanisms in the future.

To our knowledge, this is the first meta-analysis and systemic review to investigate serum vitamin D levels in AKI. However, some limitations in our study should be noted. First, since there are few studies in this area, only 4 studies with 5 cohorts were included in this paper. During study selection, one was eliminated because it is a non-quantitative description^28^, and the other cohort study of 428 people in Egypt^29^ found that 25(OH)D levels of patients in the AKI group were lower than those in the non-AKI group, presenting the results in medium (50% IQR).We attempted to contact the author for 25–75% IQR data but unfortunately did not receive a response. It is not included in our study and may could lead to bias. Secondly, all the articles in this paper are observational studies and lack of multi-center and large-cohort RCT studies, so it is difficult to determine the causal relationship. Different from animal experiments, it is very difficult to conduct large randomized controlled trials in critically ill patients due to the characteristics of clinical studies and ethical review. We can only describe the existing results for the time being and cannot draw very definite conclusions. We hope more studies will fill in the blanks in the future.

## Conclusion

The pooled estimates from the observational studies show serum 1,25(OH)2D levels, rather than 25(OH)D, is significantly lower in AKI patients when compared to non-AKI controls. The relationship between vitamin D status and clinical outcome of AKI remains controversial based on current evidence. Vitamin D is a very promising biomarker and a potential treatment for AKI. Larger studies are required to evaluate its relationship and elucidate potential mechanisms in the further.

## Supplementary Information

Supplementary Information.

## Data Availability

The datasets used and/or analyzed during the current study available from the corresponding author on reasonable request.
