# Supplementary material for: Serum vitamin D levels and acute kidney injury: a systemic review and meta-analysis
Source: Sci Rep. 2022 Nov 27;12:20365. doi: 10.1038/s41598-022-24560-4 (PMC9701671; doi:10.1038/s41598-022-24560-4)
Supplement: Supplementary file 1 — Supplementary Information. [file 41598_2022_24560_MOESM1_ESM.docx]

| **Section and Topic** | **Item #** | **Checklist item** | **Location where item is reported** |
| --- | --- | --- | --- |
| **TITLE** | | |  |
| Title | 1 | The report is identified as a mete-analysis | 1 |
| **ABSTRACT** | | |  |
| Abstract | 2 | The structured abstract includes Background, Methods, Results and Conclusion | 1 |
| **INTRODUCTION** | | |  |
| Rationale | 3 | Described in the introduction | 5 |
| Objectives | 4 | Stated in the introduction | 5 |
| **METHODS** | | |  |
| Eligibility criteria | 5 | Included and Exclusive criteria Stated in the Study selection | 7 |
| Information sources | 6 | Retrieval database and references of relevant reviews and included articles were searched manually to identify additional studies. | 6 |
| Search strategy | 7 | searched the PubMed, Web of Science and EBSCO for studies published up to 1st September 2021. Following search terms: vitamin D, 25(OH) vitamin D, 25(OH) D, 1,25(OH)2 vitamin D, 1,25(OH)2D, AKI, acute kidney injury, acute renal injury were used. ‘OR’ was used as the set operator to combine different sets of results | 7 |
| Selection process | 8 | Two investigators (HRZ and YJ) independently reviewed all identified articles for eligibility using the above criteria. The titles and abstracts of the identified articles were reviewed, and those deemed ineligible were excluded. The full text of the remainder of the articles was retrieved and reviewed. Discrepancies on whether to include a study were resolved by discussion. | 7 |
| Data collection process | 9 | The data extracted from each included study by two separate reviewers (HRZ and YJ). The data were reviewed to identify duplicate studies and duplicate reporting of populations. Only the most comprehensive studies were retained. | 7 |
| Data items | 10a | included the sample size, follow-up, the quantitative value of 25(OH)D and/or 1,25(OH)_2_D between AKI patients and non-AKI controls | 7 |
|  | 10b | included the authors, year of publication, design of the trial, sex. | 7 |
| Study risk of bias assessment | 11 | The risk of bias was evaluated as described in the Cochrance handbook by two independent reviewers(HRZ and YJ) with discrepancies discussed with a third reviewer(NS).Studies were judged as a low risk of bias if they were low risk in the following key domains. | 7 |
| Effect measures | 12 | pooled Mean Deference (MD) and 95% confidence intervals (CI) with the Inverse-Variance method using a random effects model. Heterogeneity was assessed by the I2 measure of inconsistency, statistically significant if I2 was >50%. For all the outcomes a P < 0.05 was considered statistically significant. | 8 |
| Synthesis methods | 13a | Patients were divided into groups AKI or non-AKI according to whether AKI occurred, Lai's study included two control groups, age- and gender-matched healthy subjects(h) and critically ill patients without AKI(i). Therefore, we compared these two control groups with the experimental group (AKI) separately as two cohorts, while AKI group’s sample size was divided half in each cohort. For continuous variables, | 8 |
|  | 13b | Quantitative vitamin D values in 3 of the 4 included articles were recorded in the form of mean ± standard deviation (SD), while the remaining one recorded in the form of medium (25%-75% IQR). To unify data, we transformed medium (25%-75% IQR) to mean ±SD. | 8 |
|  | 13c | The average 1,25(OH)2D levels were significant lower in AKI patients than that in non-AKI controls exhibited great heterogeneity (I2=95%, p＜0.0001). After sensitivity analysis, removing Vijiyuan et al significantly reduced this heterogeneity (I2= 0, p = 0.45), without much impact on the outcome of 1,25(OH)2D levels. | 10 |
|  | 13d | Meta-analyses were performed using Review Manager 5.3.5. | 9,10,11 |
|  | 13e | Try to find the most heterogeneous data and remove. | 10 |
|  | 13f | No more analyses conducted. | / |
| Reporting bias assessment | 14 | Since there are few studies in this area, only 4 studies with 5 cohorts were included in this paper. And the articles in this paper are observational studies and lack of multi-center and large-cohort RCT studies, so it is difficult to determine the causal relationship. | 14 |
| Certainty assessment | 15 | Since there are few studies in this area, only 4 studies with 5 cohorts were included in this paper. And the articles in this paper are observational studies and lack of multi-center and large-cohort RCT studies, so it is difficult to determine the causal relationship. | 14 |
| **RESULTS** | | |  |
| Study selection | 16a | Records after database screening  n=446 （Pubmed=152，Web of Science=138 EBSCO=156）  Records dentified through screening in the reference list（n=27）  Records afted duplicates removed  （n=128）  Records excluded  （n=108）  Full-text articles excluded(n=16)  Different ways of grouping(n=11)  Nonquantitative data(n=2)  Repetition of research(n=2)  could not reach the author（n=1）  Full-text articles assessed for eligibility（n=20）  Studies included in quantitative sysnthesls（n=4）  assessed the quality of papers（n=4）  Records screened  （n=128） | 21 |
|  | 16b | No other flow chart. | / |
| Study characteristics | 17 | The flow diagram for study selection is displayed in Fig. 1. A total of 446 records through searching the electronic database and 27 in the reference list articles identified. After removing duplicate publications, 128 studies were retrieved for title and abstract review. A total of 20 studies went further for full-text evaluation. 11 studies were excluded due to inconsistent study and grouping methods;2 were excluded because the report form cannot be used to calculate the results, 2 because repetition of research; 1 because we could not reach the author. We eliminated two overlapping data, including only the most recent or comprehensive data. Finally, four studies with five cohorts fulfilled the inclusion criteria and were suitable for the analysis. | 9 |
| Risk of bias in studies | 18 | Present data on risk of each study and, if available, any outcome level assessment | 9,10,11 |
| Results of individual studies | 19 | For all outcomes considered for each study:（a）Vitamin D values in AKI patients and non-AKI controls，（b）Relationship between vitamin D levels and prognosis of AKI. | 9,10,11 |
| Results of syntheses | 20a | The sample size of this study was small, and the bias was controlled by eliminating the study with high heterogeneity. | / |
|  | 20b | For continuous variables, we calculated the pooled Mean Deference (MD) and 95% confidence intervals (CI) with the Inverse-Variance method using a random effects model. Heterogeneity was assessed by the I2 measure of inconsistency, statistically significant if I2 was >50%. For all the outcomes a P < 0.05 was considered statistically significant. | 8 |
|  | 20c | The average 1,25(OH)2D levels were significant lower in AKI patients than that in non-AKI controls exhibited great heterogeneity (I2=95%, p＜0.0001). After sensitivity analysis, removing Vijiyuan et al significantly reduced this heterogeneity (I2= 0, p = 0.45), without much impact on the outcome of 1,25(OH)2D levels. | 10 |
|  | 20d | Heterogeneity was assessed by the I2 measure of inconsistency, statistically significant if I2 was >50%. For all the outcomes a P < 0.05 was considered statistically significant. | 8 |
| Reporting biases | 21 | since there are few studies in this area, only 4 studies with 5 cohorts were included in this paper. During study selection, one was eliminated because it is a non-quantitative description, and the other cohort study of 428 people in Egypt found that 25(OH)D levels of patients in the AKI group were lower than those in the non-AKI group, presenting the results in medium(50% IQR).We attempted to contact the author for 25%-75% IQR data but unfortunately did not receive a response. It is not included in our study and may could lead to bias. Secondly, all the articles in this paper are observational studies and lack of multi-center and large-cohort RCT studies, so it is difficult to determine the causal relationship | 14 |
| Certainty of evidence | 22 | For continuous variables, we calculated the pooled Mean Deference (MD) and 95% confidence intervals (CI) with the Inverse-Variance method using a random effects model. Heterogeneity was assessed by the I2 measure of inconsistency, statistically significant if I2 was >50%. For all the outcomes a P < 0.05 was considered statistically significant. | 8 |
| **DISCUSSION** | | |  |
| Discussion | 23a | The pooled estimates from the observational studies show serum 1,25(OH)2D levels, rather than 25(OH)D, is significantly lower in AKI patients when compared to non-AKI controls. The relationship between vitamin D status and clinical outcome of AKI remains controversial based on current evidence. | 14,15 |
|  | 23b | To our knowledge, this is the first meta-analysis and systemic review to investigate serum vitamin D levels in AKI. However, some limitations in our study should be noted. First, since there are few studies in this area, only 4 studies with 5 cohorts were included in this paper. During study selection, one was eliminated because it is a non-quantitative description. |  |
|  | 23c | The articles in this paper are observational studies and lack of multi-center and large-cohort RCT studies, so it is difficult to determine the causal relationship. Different from animal experiments, it is very difficult to conduct large randomized controlled trials in critically ill patients due to the characteristics of clinical studies and ethical review. We can only describe the existing results for the time being and cannot draw very definite conclusions. We hope more studies will fill in the blanks in the future. | 14 |
|  | 23d | The relationship between vitamin D status and clinical outcome of AKI remains controversial based on current evidence. Vitamin D is a very promising biomarker and a potential treatment for AKI.. | 14,15 |
| **OTHER INFORMATION** | | |  |
| Registration and protocol | 24a | no registration information | / |
|  | 24b | a protocol was not prepared. | / |
|  | 24c | not available | / |
| Support | 25 | This work was supported by grants from Zhejiang Provincial Natural Science Foundation of China (grant numbers: LQ19H050009) and the Foundation of Key Discipline Construction of Zhejiang Province for Traditional Chinese Medicine (No. 2017-XKA36) . | 3 |
| Competing interests | 26 | The authors declare no competing interests. | 2,15 |
| Availability of data, code and other materials | 27 | The datasets used and/or analyzed during the current study available from the corresponding author on reasonable request. | 15 |

*From:*  Page MJ, McKenzie JE, Bossuyt PM, Boutron I, Hoffmann TC, Mulrow CD, et al. The PRISMA 2020 statement: an updated guideline for reporting systematic reviews. BMJ 2021;372:n71. doi: 10.1136/bmj.n71

For more information, visit: <http://www.prisma-statement.org/>
